# Enhancements of thermal conductivities with Cu, CuO, and carbon nanotube nanofluids and application of MWNT/water nanofluid on a water chiller system

**DOI:** 10.1186/1556-276X-6-297

**Published:** 2011-04-05

**Authors:** MinSheng Liu, Mark ChingCheng Lin, ChiChuan Wang

**Affiliations:** 1 Green Energy & Environment Research Laboratories, Industrial Technology Research Institute, Hsinchu, Taiwan; 2 Material & Chemical Research Laboratories, Industrial Technology Research Institute, Hsinchu, Taiwan; 3 Department of Mechanical Engineering, National Chiao Tung University, Hsinchu, Taiwan

## Abstract

In this study, enhancements of thermal conductivities of ethylene glycol, water, and synthetic engine oil in the presence of copper (Cu), copper oxide (CuO), and multi-walled carbon nanotube (MWNT) are investigated using both physical mixing method (two-step method) and chemical reduction method (one-step method). The chemical reduction method is, however, used only for nanofluid containing Cu nanoparticle in water. The thermal conductivities of the nanofluids are measured by a modified transient hot wire method. Experimental results show that nanofluids with low concentration of Cu, CuO, or carbon nanotube (CNT) have considerably higher thermal conductivity than identical base liquids. For CuO-ethylene glycol suspensions at 5 vol.%, MWNT-ethylene glycol at 1 vol.%, MWNT-water at 1.5 vol.%, and MWNT-synthetic engine oil at 2 vol.%, thermal conductivity is enhanced by 22.4, 12.4, 17, and 30%, respectively. For Cu-water at 0.1 vol.%, thermal conductivity is increased by 23.8%. The thermal conductivity improvement for CuO and CNT nanofluids is approximately linear with the volume fraction. On the other hand, a strong dependence of thermal conductivity on the measured time is observed for Cu-water nanofluid. The system performance of a 10-RT water chiller (air conditioner) subject to MWNT/water nanofluid is experimentally investigated. The system is tested at the standard water chiller rating condition in the range of the flow rate from 60 to 140 L/min. In spite of the static measurement of thermal conductivity of nanofluid shows only 1.3% increase at room temperature relative to the base fluid at volume fraction of 0.001 (0.1 vol.%), it is observed that a 4.2% increase of cooling capacity and a small decrease of power consumption about 0.8% occur for the nanofluid system at a flow rate of 100 L/min. This result clearly indicates that the enhancement of cooling capacity is not just related to thermal conductivity alone. Dynamic effect, such as nanoparticle dispersion may effectively augment the system performance. It is also found that the dynamic dispersion is comparatively effective at lower flow rate regime, e.g., transition or laminar flow and becomes less effective at higher flow rate regime. Test results show that the coefficient of performance of the water chiller is increased by 5.15% relative to that without nanofluid.

## Introduction

Nanomaterials have been extensively researched in recent years. Emerging nanotechnology shows promise in every aspect of engineering applications. A new approach to nanoparticles in nanofluid was proposed by Choi [1], who coined the term 'nanofluid' at the USA's Argonne National Laboratory in 1995. Nanofluids are of great scientific interest because the new thermal transport phenomena surpass the fundamental limits of conventional macroscopic theories of suspensions. Furthermore, nanofluids technology can provide exciting new opportunities to develop nanotechnology-based coolants for a variety of innovative applications [2].

The thermal conductivity of heat transfer fluid plays an important role in the development of energy-efficient heat transfer equipments including electronics, HVAC&R, chemical processing, and transportation. Development of advanced heat transfer fluids is clearly essential to improve the effective heat transfer behavior of conventional heat transfer fluids. With a tiny addition of nanoparticle, significant rise of thermal conductivity is achieved without suffering considerable pressure drop penalty.

As seen, there had been considerable research and development focusing on nanofluids. Thermal conductivity enhancement for available nanofluids is shown to be in the 15 to 40% range, with a few situations reporting orders of magnitude enhancement [3]. Hwang et al. [4] measured the pressure drop and convective heat transfer coefficient of water-based Al_2_O_3 _nanofluids flowing through a uniformly heated circular tube in the full developed laminar flow regime. The enhancement of convective heat transfer coefficient is 8% which is much higher than that of effective thermal conductivity rise of 1.44% at the same volume fraction of 0.3 vol.%. However, these studies are mainly focused either on the measurement and calculation of basic physical properties like thermal conductivity and viscosity or the overall heat transfer and frictional characteristics of nanofluids.

In our previous study, different nanofluids including copper (Cu), copper oxide (CuO), and multi-walled carbon nanotube (MWNT) were synthesized for measurement of thermal conductivity. In this study, those previous results are first systematically evaluated for a better understanding for application of heat transfer medium.

Until now, there were few studies associated with the overall system performance or with field test in which some dynamic characteristics of the system may be missing. In that regard, in our previous study, the overall system performance of a 10-RT water chiller (air conditioner) subject to the influence of MWNT/water nanofluid was tested. In this study, the main purpose is to elaborate the possible mechanism for the system performance that was not studied, and to address the associated applicability for industry water chiller system along with more measured properties.

## Experiments

Nanofluids, as a kind of new engineering material consisting of nanometer-sized additives and base fluids, have attracted great attention of investigators for their superior thermal properties and many potential applications. Many investigations on nanofluids were reported, especially some interesting phenomena, new experimental results and theoretical study on nanofluids [5].

Many studies on the thermal conductivities of nanofluids had focused on the nanofluids synthesized methods such as physical mixing. In previous study, the enhancements of the thermal conductivity of ethylene glycol and synthetic engine oil in the presence of CuO nanoparticles and MWNTs were investigated using the physical mixing method [6,7]. The previous study also reported the chemical reduction method for synthesis of nanofluids containing Cu nanoparticles in water [8].

In previous study, CuO nanofluids were prepared by the physical mixing method (two-step method) [6]. First, CuO nanoparticles were prepared. Nonmetal CuO nanoparticles were produced by a physical vapor synthesis method (Nanophase Technologies Corp., Romeoville, Illinois, USA). The CuO powders were then dispersed into the ethylene glycol base fluid. The average particle size of CuO powders was 29 nm as received. MWNTs nanofluids were also prepared using the physical mixing method [7]. MWNTs were prepared first. MWNTs were produced by catalytic chemical vapor deposition method (Nanotech Port Co., Shenzhen, China).

After being mixed in the ethylene glycol base fluid, CuO solid nanoparticles were dispersed by magnetic force agitation; the suspensions were then homogenized by intensive ultrasonics. Stable nanofluids were successfully prepared without adding surfactants. MWNTs were then added to ethylene glycol or synthetic engine oil base fluids. No surfactant was used in MWNT-ethylene glycol suspensions. *N*-hydroxysuccinimide (NHS) was, however, employed as the dispersant in MWNT-synthetic engine oil suspensions. NHS was in the solid particle form. NHS was added into carbon nanotubes (CNTs) directly.

On the other hand, the chemical reduction method (one-step method) was used to synthesize Cu nanoparticles in the presence of water as solvent under nitrogen atmosphere in previous study [8]. Copper acetate (Cu(CH_3_COO)_2_) was used as the precursor. Hydrazine (N_2_H_4_) acted as a reducing agent. No surfactant was employed as the dispersant. The copper acetate was dissolved in deionized (D.I.) water. The solution was stirred uniformly at a temperature of 55°C under nitrogen.

The Cu and CuO nanoparticles were measured with scanning electron microscopy (SEM) to determine their microstructure. MWNTs were also measured with SEM and high-resolution transmission electron microscopy (HRTEM) to determine their microstructure. On the preparation of those nanomaterials for SEM, those nanomaterials are coated with gold (Au) and palladium (Pd) to increase the electrical conductivity before sent to vacuum chamber of SEM. Therefore, the coating layers are Au and Pd.

The most commonly used technique for measuring thermal conductivity of nanofluids is the transient hot wire technique. This measurement technique has gained popularity because the thermal conductivity of the liquid can be measured instantaneously with a good level of accuracy and repeatability [9].

**Figure 1:**
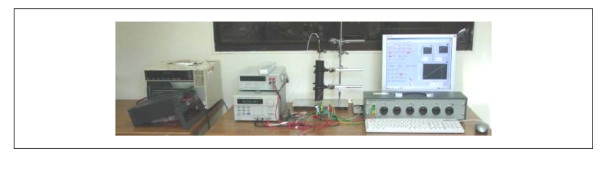
**The modified computer-controlled hot wire system for measurement of thermal conductivity**.

A modified computer-controlled hot wire system has been designed for the measurement of thermal conductivity of nanofluids. The apparatus used is shown in Figure 1.

For the transient hot wire system, a thin platinum wire was immersed in the fluid using a vertical cylindrical glass container. The hot wire served as an electrical resistance thermometer. A Wheatstone bridge heated the platinum wire and simultaneously measured its resistance. The electrical resistance of the platinum wire varies in proportion to changes in temperature. The thermal conductivity was then estimated from Fourier's law. The nanofluids were filled into the glass container to measure the thermal conductivity. The inner diameter and length of long glass container are 19 and 240 mm, respectively. The transient hot wire system was calibrated with D.I. water and ethylene glycol at room temperature. Uncertainty of the measurement is less than 2%.

The viscosity is measured with portable viscosimeter with deviation being less than 1% (Hydramotion VL700). The specific heat of MWNT/water nanofluid was also measured using differential scanning calorimetry (DSC) (TA Instrument 5100). The test condition of DSC was that equilibrates at -10°C, isotherm for 5 min, ramp 10°C/min to 90°C, and isotherm for 5 min.

Furthermore, the comparison of heat transfer behavior of a water chiller cooling system between the pure water and nanofluid was made [10]. MWNT/water nanofluids were prepared using two-step method as described previously. MWNTs powders were added to water base fluid. The city water (tap water) was used due to the large amount of water is needed for a 10-RT water chiller. The volume fraction of MWNT/water was 0.001 (0.1 vol.%) and the thermal conductivity was increased up to 1.3% at room temperature without surfactant and surface treatment. The addition of dispersant and surfactant would make the MWNT coated and result in the screening effect on the heat transfer performance of MWNT. Furthermore, the MWNT nanofluid could be agitated continuously to achieve good dispersion dynamically when the pump of test system is driving.

**Figure 2:**
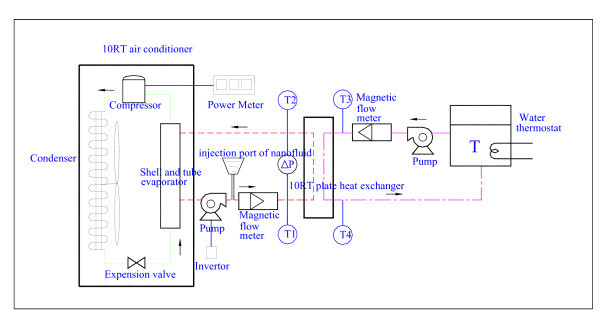
**Schematic diagram of the experimental test system for the water chiller with a nominal 10-RT capacity using MWNT/water nanofluid**.

In previous study, the system performance of a water chiller (air conditioner) with 10-RT capacity was conducted at a well-controlled environment chamber. Figure 2 shows a schematic diagram of the experimental test system for the water chiller with a nominal 10-RT capacity. Tests were conducted with and without the addition of MWNT/water nanofluid.

The test system included a base fluid loop and a water loop. The base fluid could be supplied with either water or with nanofluid; it consisted of an air-cooled chiller, a forced circulation pump for delivering chilled water being generated, an injection port of nanofluid, and a plate heat exchanger, a water thermostat with 6000-L capacity, MWNT/water nanofluid, and measuring devices. The air-cooled chiller included a compressor, a power meter, a fin-and-tube air-cooled condenser, a shell-and-tube evaporator, and an expansion valve. R-22 was the working refrigerant for the air-cooled chiller. The water loop was used to consume the chilled water being produced from the air-cooled chiller via a plate heat exchanger. The flow rate of base fluid was controlled by the inverter. The water tubing into the test plate heat exchanger was made of stainless steel tube with an outer diameter of 32-mm and an inside diameter of 25.4-mm.

On the other hand, a water loop was designed to balance the chilled water energy from the air-cooled chiller, containing a circulation pump and a water thermostat. The component and piping of system were well insulated with respect to the surrounding environment.

The temperature sensor and pressure sensor were used to monitor the fluid temperature and pressure at various locations. Calibrated RTDs (resistance temperature detector) with 0.02°C accuracy were used to measure the inlet and outlet temperature of each water loop. Differential pressure transducer was used to measure the pressure difference of the refrigerant loop. The maximum pressure difference (Yakogawa EJA110A) is as high as 10000 mm H_2_O, and the corresponding maximum uncertainty is less than 2.4%. The maximum flow rate of magnetic flowmeter is 300 L/min. The power meter was used to monitor the consumed electric power. All the measuring devices were precalibrated. Furthermore, all the data signals were collected via the data acquisition system connected to a personal computer. The data acquisition system included a hybrid multipoint recorder (Yakogawa DR230), a power distributor, a NI GPIB interface, and a personal computer. The measured cooling capacity and consumed electric power could be used to calculate the overall system performance subject to the addition of nanofluid. The uncertainty of the measured cooling capacity of the test span ranges from ±0.9 to ±1.1%. The highest uncertainty occurs at the maximum flow rate of 140 L/min.

The system performance of the air-cooled chiller was conducted in a well-controlled environment chamber capable of maintaining a controlled environment to meet the requirements of ARI 550/590 (standard for water chilling packages using the vapor compression cycle). The standard outdoor conditions were 35°C (dry bulb) and 24°C (wet bulb), whereas the indoor ambient was fixed at 27°C (dry bulb) and 19°C (wet bulb). The maximum temperature deviation was within 0.05°C and the airflow uniformity of within the environment chamber was less than 0.05 m/s. Following the standard test of chiller, the test was first performed with the standard water chiller rating condition: water inlet temperature at 7°C (*T*_1_), water outlet temperature at 12°C (*T*_2_), and at a flow rate of 85 L/min.

Tests were performed for comparisons between water base fluid and MWNT/water nanofluid. In the first run, the water base fluid was used as the heat transfer medium in the evaporator. The outlet temperature of the heat exchanger was maintained at 12°C (*T*_2_). The inlet temperature at left-hand side of the plate heat exchanger (*T*_1_) shown in Figure 2 was varying in association with the flow rate from 80 to 140 L/min. In the second run, the nanofluid (MWNT/water nanofluid) was used for testing. Ranges of the flow rate are from 60 to 140 L/min at interval of 20 L/min. The inlet temperature of cooling water was maintained at 14°C (*T*_3_) by a water thermostat. The outlet temperature (*T*_4_) of the plate heat exchanger was also changing under the variations of the flow rate from 80 to 140 L/min at interval of 20 L/min.

In order to gain a good control on the stability of flow rate, the inverter-fed pump was used. The electric power of circulation pump and inverter was supplied externally by an independent power source and was thus not counted in the consumed electric power of experimental water chiller test system. The consumed electric power included compressor, fan of condenser, and the controller.

The experimental result regarding the heat transfer performance of nanofluid for a water chiller thus could provide an example on the nanofluid behavior in industry thermal application.

## Results and discussion

The thermal conductivity of heat transfer fluid is of great consequence in the improvement of energy-efficient heat transfer. It is clearly needed to develop advanced heat transfer fluids for improving the effective heat transfer behavior of conventional heat transfer fluids.

**Figure 3:**
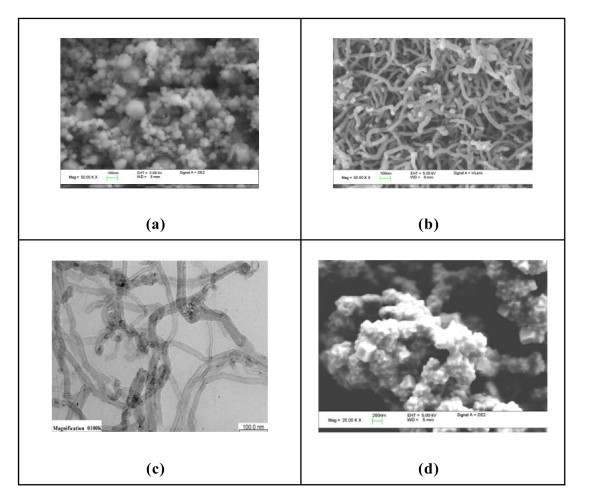
**Typical SEM micrographs and HRTEM micrograph of CuO, MWNT, and Cu**. **(a) **Typical SEM micrograph of CuO nanoparticles; **(b) **typical SEM micrograph of MWNTs; **(c) **typical HRTEM micrograph of MWNTs; **(d) **typical SEM micrographs of Cu nanoparticles.

Typical SEM micrograph of CuO nanoparticles is shown in Figure 3a. The morphology and particle size of CuO powders are clearly seen. The CuO powders generally exhibit small particle sizes and a narrow distribution. The agglomerated CuO nanoparticles range from 30 to 50 nm with spherical shape. A typical SEM micrograph of MWNTs is shown in Figure 3b. The randomly oriented fiber-like MWNTs are clearly seen. An individual MWNT is several microns long. Small catalytic, metallic nanoparticles are observed at the tip of the MWNT with diameters of 20 to 30 nm. Figure 3c shows a typical HRTEM micrograph of MWNTs. The HRTEM image clearly shows the characteristic features of MWNTs. The MWNT core is hollow with multiple layers almost parallel to the MWNT axis. Its inner diameters are about 5 to 10 nm, and outer diameters are about 20 to 50 nm, respectively. Typical SEM micrograph of Cu nanoparticles is shown in Figure 3d. Cu nanoparticles synthesized by chemical reduction shows the monodispersed distribution of particle sizes. The agglomerated particle sizes of the Cu nanoparticles range from 50 to 100 nm with spherical and square shapes.

**Figure 4:**
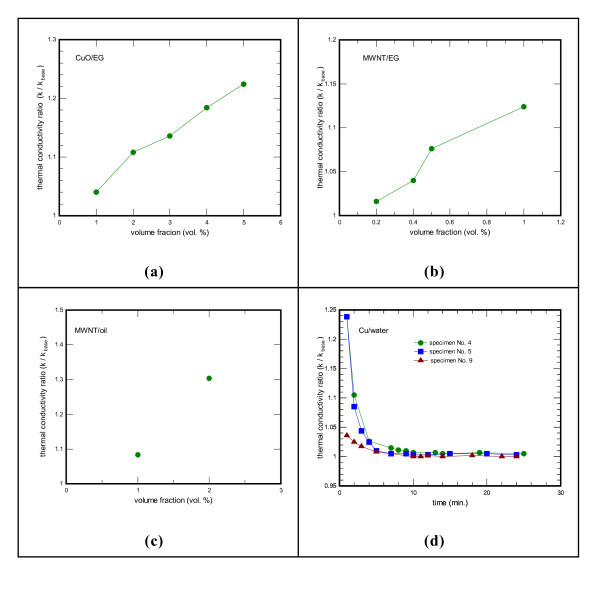
**The normalized thermal conductivity of Cu, CuO, and MWNT nanofluids as a function of the volume fraction**. **(a) **The normalized thermal conductivity of CuO-ethylene glycol nanofluids as a function of volume fraction; **(b) **the normalized thermal conductivity of MWNT-ethylene glycol nanofluids as a function of volume fraction; **(c) **the normalized thermal conductivity of MWNT-synthetic engine oil nanofluids as a function of volume fraction; **(d) **the normalized thermal conductivity of Cu-water nanofluids as a function of the measured time at 0.1 vol.%.

Figure 4 shows the normalized thermal conductivity of Cu, CuO, and MWNT nanofluids as a function of the volume fraction. The *k *is the thermal conductivity of nanoparticles suspensions and the *k*_base _is the thermal conductivity of the base fluid. The thermal conductivity ratio enhancements of CuO and MWNT nanofluids increase with the increase of volume fraction of CuO and MWNT. The thermal conductivity ratio improvement for CuO nanofluid is approximately linear with the nanoparticle volume fraction (Figure 4a). For CuO nanoparticle at a volume fraction of 5 vol.% dispersed in ethylene glycol, thermal conductivity enhancements up to 22.4% are observed. Thermal conductivity enhanced by 22% at 4 vol.% has been reported for CuO-ethylene glycol suspensions [11].

The results for MWNT nanofluid with different volume fractions also exhibit the same trend (Figure 4b, c). For MWNT-ethylene glycol suspensions at 1 vol.%, thermal conductivity enhancements of up to 12.4% are observed. On the other hand, for MWNT-synthetic engine oil suspension, thermal conductivity is enhanced by 30% at a volume fraction of 2 vol.%. For MWNT-ethylene glycol suspension, thermal conductivity enhanced by 12.7% at 1 vol.% has been reported [12]. Moreover, for MWNT-synthetic poly oil suspensions, the measured enhancement in thermal conductivity with 1 vol.% nanotubes in oil is 160% as reported previously [13].

Cu-water nanofluids with a low concentration of nanoparticles have considerably higher thermal conductivities than the identical water base liquids without solid nanoparticles (Figure 4d). A strong dependence of thermal conductivity on the measured time is observed. In addition, at a constant volume fraction, *k*/*k*_base _is the largest at the starting point of measurement and drops considerably with elapsed time. For Cu nanoparticles at 0.1 vol.%, thermal conductivity is enhanced by 23.8%. The ratio of *k*/*k*_base _is almost unchanged when the elapsed time is above 10 min. The value of *k*/*k*_base _is slightly above unity, indicating no appreciable enhancements due to particles agglomeration. The volume fractions of Cu nanoparticles suspended in water are 0.1 vol.% for specimens no. 4 and no. 5 and 0.2 vol.% for specimens no. 9, respectively. Xuan and Li [14] showed that the ratio of the thermal conductivity of the Cu-water nanofluid to that of the base liquid varies from 1.24 to 1.78 when the volume fraction of the nanoparticles increases from 2.5 to 7.5 vo1.%. The corresponding Cu nanoparticles were about 100 nm diameter and were directly mixed with D.I. water. The laurate salt at several weight percents was used to enhance stability of the suspension. Furthermore, the tendency of the settlement time dependence of thermal conductivity enhancements is also reported in ethylene glycol-based Cu nanofluids [15].

Recently, Jiang and Wang [16] developed a novel one-step chemical reduction method to fabricate nanofluids with very tiny spherical Cu nanoparticles. The particle size varies from 6.4 to 2.9 nm by changing the surfactant concentration. The thermal conductivity measurement shows the existence of a critical particle size below which the nanoparticles cannot significantly enhance fluid conductivity due to the particle conductivity reduction and the solid-liquid interfacial thermal resistance increase as the particle size decreases. By considering these two factors, the critical particle size is predicted to be around 10 nm based on theoretical analysis. In present study, Cu-water nanofluids are also synthesized using chemical method but without surfactant. The agglomerated particle sizes of the Cu nanoparticles range from 50 to 100 nm with spherical and square shapes.

The typical value of thermal conductivity is 0.25 W/m K for ethylene glycol, 0.6 W/m K for water, 33 W/m K for CuO, 400 W/m K for Cu, and 2000 W/m K for MWNT [12]. There are three orders of magnitude difference between liquids and solid particles for thermal conductivity. Therefore, fluids containing solid particles can be anticipated to show appreciably enhanced thermal conductivities compared with pure fluids. The thermal conductivity of MWNT/ethylene glycol nanofluid is increased by about 12.4% at 1 vol.% as shown in Figure 4b. The high conductivity and high aspect ratio of MWNT make it especially suitable for heat transfer in a nanofluid. Furthermore, MWNT can also act as a lubricating medium due to its small size. In this study, the MWNT is thus used as the heat transfer medium for a 10-RT water chiller.

Heat transfer takes place on the surface of the solid particles. In this study, SEM shows very narrowly size-distributed Cu and CuO nanoparticles and MWNT. Compared with conventional particles, nanoparticles accommodate much larger surface areas per volume. For example, the surface area to volume ratio (*A*/*V*) is 1000 times larger for particles in 10 nm diameter than in 10 μm diameter [11]. The larger surface area can thus increase heat transfer capabilities [17]. Fluids with solid particles on a nano scale show better thermal conductivities than fluids with coarse solid particles on a micro scale. This is associated with large total surface areas of nanoparticles.

The viscosity is measured with portable viscosimeter. The viscosity of CuO nanofluids is also found to increase with the volume ratio. It is seen that the viscosity is increased by 10.7% at a volume fraction of 0.01 (1 vol.%) and up to 83.4% at 5 vol.%. The thermal conductivity property is enhanced by the presence of CuO nanofluids. On the other hand, the increase of viscosity may offset the benefit from enhanced thermal conductivity. Optimum conditions between thermal conductivity and viscosity of CuO nanofluids need to be taken into consideration in heat transfer applications.

The measured viscosity of tap water (city water) is 0.8 cps at 23.5°C and that of MWNT/tap water nanofluid is 1.0 cps at 24.1°C. It is thus expected that the slight increased viscosity of MWNT nanofluid would only cast minor impact on the pumping power of heat transfer system.

**Figure 5:**
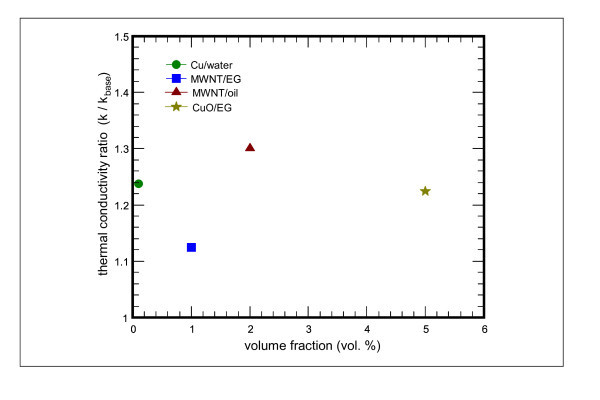
**The normalized thermal conductivity as a function of volume fraction for the Cu, CuO, and MWNT nanofluids**.

Figure 5 shows a plot of normalized thermal conductivity as a function of volume fraction for Cu, CuO, and MWNT nanofluids. The thermal conductivity enhancement is found to be of different order at different volume fraction. From this figure, one also can see that a notable difference exists for measured thermal conductivity ratios with the addition of different nanoparticles.

For practical applications of nanofluids, a constructal approach is proposed by Wang and Fan [18] recently. It is based on the constructal theory to convert the inverse problem of nanofluid microstructural optimization into a forward one by first specifying a type of microstructures and then optimizing system performance with respect to the available freedom within the specified type of microstructures, and enables us to find the constructal microstructure. That is the best for the optimal system performance within the specified type of microstructures.

In Meibodi et al.'s recent work [19], the effects of different factors on thermal conductivity and stability of CNT/water nanofluids, including nanoparticle size and concentration, surfactant type and concentration, pH, temperature, power of ultrasonication and elapsed time after ultrasonication, and their interactions have been investigated experimentally. The most suitable condition for production and application of CNT/water nanofluid has been proposed based on statistical analysis of the results. It has been shown that more stable nanofluid may not necessarily have higher value of thermal conductivity. Thermal conductivity of nanofluid is time dependent immediately after ultrasonication and independent of time at longer time. In our present study, stable CNT nanofluid is successfully obtained.

For the industrial application of nanofluid on cooling, the nanofluid can be used for refrigerant medium of air conditioning and refrigeration (AC&R). The nano-refrigerant is one kind of nanofluid with host fluid being refrigerant. A nano-refrigerant has higher heat transfer coefficient than the host refrigerant and it can be used to improve the performance of refrigeration systems. Jiang et al. [20] recently reported on the experimental results show that the thermal conductivities of CNT nano-refrigerants are much higher than those of CNT-water nanofluids or spherical-nanoparticle-R113 nano-refrigerants. The thermal conductivities of CNT nano-refrigerants increase significantly with the increase of the CNT volume fraction. When the CNT volume fraction is 1.0 vol.%, the measured thermal conductivities of four kinds of CNT-R113 nano-refrigerants are increased by 82, 104, 43, and 50%, respectively. The thermal conductivity enhancements of CNT-R113 nano-refrigerants are higher than those of CNT-water nanofluids and spherical nanoparticles-R113 nano-refrigerants with the same nanoparticle volume fraction.

For the application of nanofluid on heat transfer device, the performance of a commercial herringbone-type plate heat exchanger using 4 vol.% CuO nanofluid is experimentally studied by Pantzali et al. [21]. Prior to this heat exchanger, the thermophysical properties of several nanofluids including CuO, Al_2_O_3_, TiO_2_, and CNT in water were systematically measured. The general trends of nanofluids including increase of thermal conductivity, density, viscosity, and decrease of heat capacity are confirmed. Besides the physical properties, the flow regime (laminar or turbulent) inside the heat exchanger also affects the efficiency of a nanofluid as coolant. The fluid viscosity seems also to be an important factor. It is concluded that turbulent flow, which is commonly employed in this industrial heat exchanger, normally requires large volumetric concentration of nanofluids. Hence the replacement of conventional fluids by nanofluids may cause additional concerns like clogging, sedimentation, and wearing for fluid machineries.

Nanofluids with cylindrical CNT generally show greater thermal conductivity enhancement than nanofluids with spherical particles. This might be due to the rapid heat transport along relatively larger distances in cylindrical particles since cylindrical particles usually have lengths on the order of micrometers. However, nanofluids with cylindrical particles usually have much larger viscosities than those with spherical nanoparticles [22]. In present study, the volume fraction of MWNT/water used is only 0.001 (0.1 vol.%) and the relevant increase in thermal conductivity is only up to 1.3% at room temperature condition. The measured viscosity of tap water is 0.8 cps at 23.5°C and that of MWNT/tap water nanofluid is 1.0 cps at 24.1°C. Note that there is no surfactant or dispersant used for the nanofluids. It is thus expected that the associated increase in pumping power is small and this increases the potential usage of MWNT nanofluids in heat exchanger system.

**Figure 6:**
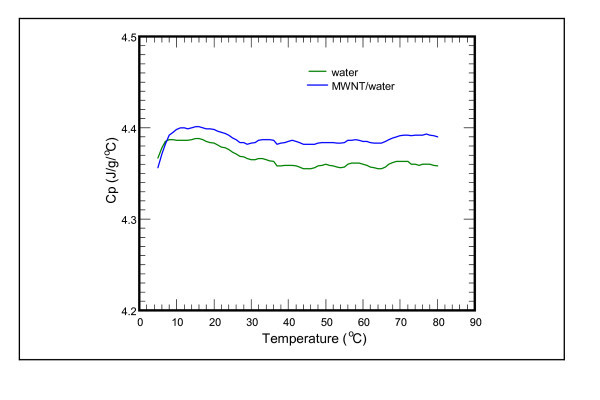
**Specific heat vs. temperature subject to the influence of MWNT/water nanofluid at 0.1 vol.%**.

In addition to thermal conductivity, the specific heat also affects the performance of nanofluid. The specific heat of city water (tap water) is 4.383 J/g K at 20°C (4.373 J/g K at 25°C). The specific heat of D.I. water is 4.456 J/g K at 20°C (4.454 J/g K at 25°C). The specific heat of MWNT is 0.6 J/g K at 20°C. On the other hand, the specific heat of MWNT/city water nanofluid is 4.398 J/g K at 20°C (4.389 J/g K at 25°C). Therefore, the specific heat of MWNT/city water nanofluid at 0.1 vol.% is higher than that of city water. The specific heat is increased to be about 0.4% at 20°C shown in Figure 6. This indicates that the total amount of heat that can be absorbed by MWNT/city water is increased. However, the specific heat of MWNT nanofluid at 0.1 vol.% is lower than that of D.I. water. It is generally observed that the heat capacity is decreased with the addition of nanoparticles. From the measured experimental data for CuO nanofluids, Zhou et al. [23] also reported that the specific heat capacity of CuO nanofluid decreases gradually with increasing volume concentration of nanoparticles.

The standard test of chiller is performed with standard water chiller rating condition: water inlet temperature at 7°C (*T*_1_), water outlet temperature at 12°C (*T*_2_), and at a flow rate of 85 L/min. For the temperature dependence of thermal conductivity with temperature, Ding et al. [24] showed that the effective thermal conductivity increases with increasing temperature in CNT-water suspensions. For a 1 wt% of MWNT/water nanofluid, 80% enhancement of thermal conductivity is achieved at 30°C while that of down to 10% is observed at 20°C. Zhang et al. [25] also showed that the thermal conductivity of the Al_2_O_3_/water nanofluid increases with an increase of the particle concentration and with the temperature. Conversely the pure water shows consistent temperature dependence tendency. In the present study, the linear relationship between thermal conductivity and temperature is used to estimate the variation of thermal conductivity with temperature. For the present study, this indicates that the increase of thermal conductivity for the MWNT/water at standard chiller rating condition is even lower than 1.3% at room temperature. Following an estimation of the linear relationship, barely enhancement of thermal conductivity is encountered (0.9%) at 10°C.

**Figure 7:**
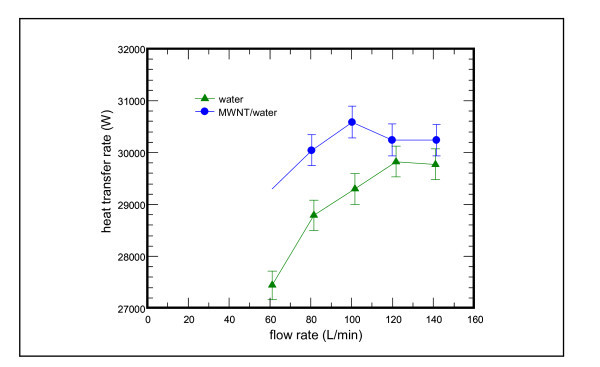
**Cooling capacity vs. flow rate subject to the influence of MWNT/water nanofluid at 0.1 vol.%**.

Cooling capacity vs. flow rate subject to the influence of nanofluids is shown in Figure 7. For the water base fluid, the cooling capacity increases with the rise of flow rate from 60 to 120 L/min. The cooling capacity, however, does not change as flow rate is further increased to 140 L/min. On the other hand, for MWNT/water nanofluid, the cooling capacity shows a similar trend but reveals an early level-off when the flow rate is increased over 100 L/min. The cooling capacity reaches a maximum value at a flow rate of 100 L/min. The effective mean flow velocity within the channel of the plate heat exchanger is about 4.5 m/s and the corresponding *Re *number is approximate 13,500 at a flow rate of 100 L/min. The flow is thus in turbulent condition. On the other hand, at a flow rate of 60 L/min, the flow velocity is about 2.7 m/s and the corresponding *Re *number is approximately 8,100. The flow is also in transition to turbulent flow.

From the comparison of cooling capacity rate between water base fluid and MWNT/water nanofluid, one can see that the cooling capacity of MWNT/water nanofluid is higher than that of water base fluid over the entire testing range. The increased cooling capacity spans 2 to 6%. The maximum difference occurs at the smallest flow rate at 60 L/min. The results are quite surprising for the foregoing measurement of thermal conductivity, for MNWT/water nanofluid shows only marginal increase in thermal conductivity (1.3% at room temperature and 0.9% at 10°C rating condition) of nanofluid relative to that of pure water, whereas the maximum capacity difference shown in Figure 7 is increased over 6%. Hence, certain dynamic characteristics of nanofluids must be in presence. One of the possible dynamic effects caused by the nanofluids is associated with dispersion effect of the nanoparticles as it flows along the heat transfer channel. For a laminar flow, the presence of nanoparticles may well distort the convectional parabolic profile, leading to an effective increase of heat transfer performance. On the other hand, though the well-dispersed nanoparticles still play an essential role for heat transfer enhancement for turbulent flow, it should be emphasized that the major thermal resistance for turbulent flow lies in the laminar sub-layer, which is nearby the heat transfer surface. As a consequence, one can see that a much larger performance augmentation is seen at a lower flow rate (60 L/min). Conversely, the capacity reaches a plateau at higher flow regime. The test results suggest that the dynamic effect of nanofluids may be more effective in the lower flow rate region, e.g., transition or laminar flow.

Similar results are also reported by Ding et al. [24] who studied the heat transfer performance of CNT nanofluid in a tube with 4.5 mm inner diameter. They found that the observed enhancement of heat transfer coefficient is much higher than that of the increase in effective thermal conductivity. They postulated several possible reasons with the abnormal increase of heat transfer coefficient, i.e., shear-induced enhancement in flow, reduced boundary layer, particle rearrangement, and high aspect ratio of CNT. These observations suggest that the aspect ratio should be associated with the high enhancement of heat transfer performance of CNT-based nanofluids.

Apart from the foregoing explanations of the possible causes, one should be aware that the measurement of thermal conductivity is performed under static condition, whereas the measurement of cooling capacity is carried out at dynamic fluid flow condition. Hence, interactions of the flow field with nanopowders may be another reason for substantial rise of cooling capacity. A recent numerical investigation concerning with the fluid flow behaviors of nanofluid via a two-phase approach was conducted by Behzadmehr et al. [26], they had clearly shown that the presence of nanopowder can absorb the velocity fluctuation energy and reduce the turbulent kinetic energy as well. However, this phenomenon becomes less pronounced when the Reynolds number is further increased. This is due to the fact that the corresponding velocity profiles become more uniform as the Reynolds number is increased. In that sense, one can see the difference in cooling capacity is reduced between nanofluid and the base fluid when the flow rate is increased.

The viscosity of water and MWNT nanofluid decreases with the increasing of temperature. The measured viscosity of tap water is 0.8 cps at 23.5°C and that of MWNT nanofluid is 1.0 cps at 24.1°C. On the other hand, Wensel et al. [27] also reported that the nanofluid of CNT with very low loading around 0.01 vol.% is very stable and the viscosity remains approximately the same as water.

**Figure 8:**
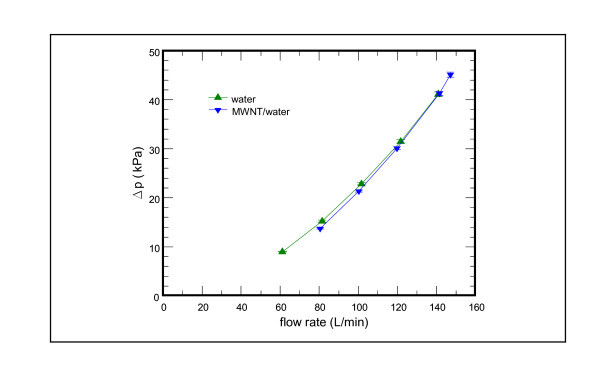
**Pressure drop vs. flow rate subject to the influence of MWNT/water nanofluid at 0.1 vol.%**.

The associated pressure drop vs. flow rate for the nanofluid and base fluid is shown in Figure 8. For both the water base fluid and the MWNT/water nanofluid, the pressure drop increases with the increase of flow rate from 60 to 150 L/min. However, negligible difference in pressure drop amid the MWNT/water nanofluid and the base fluid water is seen. The results are in line with the calculation made by Behzadmehr et al. [26]. Their two-phase modeling shows that adding 1% nanopowder results in an increase of the Nusselt number by more than 15% without appreciable increase of pressure drop. It is attributed to the absorption of turbulence caused by the nanopowders. Furthermore, Lu et al. [28] also reported that a novel and stable CNT/polystyrene hybrid miniemulsion is used as a water-based lubricant additive. The anti-wear performance and load-carrying capacity of the base stock are significantly raised and the friction factor is decreased. As a consequence, the present nanofluid with MWNT reveals negligible pressure drop penalty pertaining to the system performance of the water chiller.

Moreover, at the standard rating condition (water inlet temperature at 7°C (*T*_1_), water outlet temperature at 12°C (*T*_2_), and at a flow rate of 85 L/min), it is found that the consumed power of the nanofluid system is reduced up to 0.8% and the coefficient of performance (COP) is increased by 5.15%. The increase of COP is mainly related to the increase of cooling capacity. This is because the rise of cooling capacity inevitably slightly increases the low-side refrigeration pressure, leading to a very minor reduction in system power consumption (0.8%). In essence, the system COP is increased by 5.15% at standard rating condition.

Although the enhancement of the thermal conductivity is only up to 1.3% at room temperature and that of the specific heat is increased to be about 0.4% at 20°C. However, the increase of overall cooling capacity is about 4.2%. Moreover, Ding et al. [24] investigated the heat transfer performance of CNT nanofluids in a tube with 4.5 mm inner diameter. The observed enhancement of heat transfer coefficient is much higher than the increase in the effective thermal conductivity. It is likely that the improved heat transfer is associated with shear-induced enhancement in flow, reduced boundary layer, particle rearrangement, and high aspect ratio of CNT.

A theoretical model for the role of dynamic nanoparticles in nanofluid has been proposed [29]. The fundamental difference between solid/solid composites and solid/liquid suspensions is identified. This model is very helpful for nanofluids in the industrial applications of high efficiency heat transfer. Similar to the nanoparticles, the characteristics of interface between CNT solid and base liquid need to be exploited for better understanding of the role of CNT on the nanofluid. Therefore, fluid dynamic and convection play an important role in the enhancement of overall cooling capacity.

Recently, Pantzali et al. [21] conducted the experimental study of a commercial heat exchanger using CuO nanofluid and found that the use of nanofluid is beneficial if and only if the increase in its thermal conductivity is accompanied by a marginal increase in viscosity when the heat exchanger operate under turbulent condition. On the other hand, the use of nanofluid seems more advantageous if the heat exchanger operates under laminar condition. The results are actually in line with the foregoing discussion about the dynamic effect of nanoparticle dispersion.

In this study, the system COP of a water chiller is increased by 5.15% at standard rating condition and thus deserves further intense study for practical application in air conditioning and refrigeration industry.

## Conclusions

In our previous study, different nanofluids including Cu, CuO, and MWNT were synthesized for measurement of thermal conductivity. In this study, those results are systematically evaluated for the better application of heat transfer medium.

Until now, there were few studies associated with the overall system performance or with field test in which some dynamic characteristics of the system may be missing. In that regard, in our previous study, the overall system performance of a 10-RT water chiller (air conditioner) subject to the influence of MWNT/water nanofluid was tested. In this study, the main purpose is to elaborate some possible mechanisms for the augmentation of system performance of industry water chiller system along with more measured properties.

This study systematically evaluates the enhancements of thermal conductivities of ethylene glycol, water, and synthetic engine oil in the presence of Cu, CuO, and MWNT for the better application of heat transfer medium. The MWNT shows more promising thermal conductivity enhancement and MWNT is thus used as the heat transfer medium for the 10-RT water chiller system. This study further elaborates the possible mechanisms for the system performance of this industry water chiller system with the addition of MWNT/tap water nanofluid at 0.1 vol.%. The test system is an air-cooled water chiller with a nominal capacity of 10-RT. The increase of thermal conductivity of the nanofluid relative to the base fluid is only 1.3% at room temperature. However, the cooling capacity of the nanofluid is increased by 4.2% at the standard rating condition. The increase in cooling capacity of the nanofluid is due to dynamic interaction of the flow field and the MWNT. One of the possible causes for the considerable rise of system performance is due to the dynamic dispersion of the nanoparticles on the flow field. It is also found that the dynamic dispersion is comparatively effective at lower flow rate regime, e.g., transition or laminar flow and becomes less effective at higher flow rate regime. At the standard rating condition, the addition of nanofluid can increase the COP by 5.15% relative to that without nanofluid.

## Abbreviations

CNT: carbon nanotube; COP: coefficient of performance; D.I.: deionized; DSC: differential scanning calorimetry; HRTEM: high-resolution transmission electron microscopy; MWNT: multi-walled carbon nanotube; NHS: *N*-hydroxysuccinimide; SEM: scanning electron microscopy.

## Competing interests

The authors declare that they have no competing interests.

## Authors' contributions

All authors contributed equally.
